# Client characteristics and acceptability of a home-based HIV counselling and testing intervention in rural South Africa

**DOI:** 10.1186/1471-2458-12-824

**Published:** 2012-09-25

**Authors:** Reshma Naik, Hanani Tabana, Tanya Doherty, Wanga Zembe, Debra Jackson

**Affiliations:** 1 South African Medical Research Council, Francie van Zijl Drive, Parrowvallei, Tygerberg, 7505, South Africa; 2 Boston University, School of Public Health, 715 Albany Street, Boston, MA, 02118, USA; 3 Karolinska Institutet, Division of Global Health (IHCAR), Department of Public Health Sciences, Nobels vag 9 5-171 77, Stockholm, Sweden; 4 University of the Western Cape, School of Public Health, Modderdam Road, Bellville, 7535, South Africa

**Keywords:** Home-based HIV counselling and testing, Community-based, Acceptability, South Africa, Lay counsellors

## Abstract

**Background:**

HIV counselling and testing (HCT) is a critical gateway for addressing HIV prevention and linking people to treatment, care, and support. Since national testing rates are often less than optimal, there is growing interest in expanding testing coverage through the implementation of innovative models such as home-based HIV counselling and testing (HBHCT). With the aim of informing scale up, this paper discusses client characteristics and acceptability of an HBHCT intervention implemented in rural South Africa.

**Methods:**

Trained lay counsellors offered door-to-door rapid HIV testing in a rural sub-district of KwaZulu-Natal, South Africa. Household and client data were captured on cellular phones and transmitted to a web-based data management system. Descriptive analysis was undertaken to examine client characteristics, testing history, HBHCT uptake, and reasons for refusal. Chi-square tests were performed to assess the association between client characteristics and uptake.

**Results:**

Lay counsellors visited 3,328 households and tested 75% (5,086) of the 6,757 people met. The majority of testers (73.7%) were female, and 57% had never previously tested. With regard to marital status, 1,916 (37.7%), 2,123 (41.7%), and 818 (16.1%) were single, married, and widowed, respectively. Testers ranged in age from 14 to 98 years, with a median of 37 years. Two hundred and twenty-nine couples received couples counselling and testing; 87.8%, 4.8%, and 7.4% were concordant negative, concordant positive, and discordant, respectively. There were significant differences in characteristics between testers and non-testers as well as between male and female testers. The most common reasons for not testing were: not being ready/feeling scared/needing to think about it (34.1%); knowing his/her status (22.6%), being HIV-positive (18.5%), and not feeling at risk of having or acquiring HIV (10.1%). The distribution of reasons for refusal differed significantly by gender and age.

**Conclusions:**

These findings indicate that HBHCT is acceptable in rural South Africa. However, future HBHCT programmes should carefully consider community context, develop strategies to reach a broad range of clients, and tailor intervention messages and services to meet the unique needs of different sub-groups. It will also be important to understand and address factors related to refusal of testing.

## Background

HIV counselling and testing (HCT) is recognized as a critical gateway for addressing HIV prevention, as well as linking people to life-saving treatment, care, and support services [1-3]. However, national testing rates remain less than optimal in many affected countries. For example, in South Africa, where the national adult HIV prevalence is 17.8% [4], a 2008 survey indicates that only 24.7% of those 15–49 years had an HIV test in the past 12 months and knew their results [5]. The success of national HIV/AIDS programmes will thus necessitate the expansion of HIV testing coverage through the implementation of innovative facility- and community-based models such as home-based HIV counselling and testing (HBHCT) [6,7].

HBHCT has been studied in various sub-Saharan African contexts. Findings from cross-sectional surveys [8,9] and intervention programmes [10-15], as well as index client [16,17] and comparative study contexts [18-21], indicate the feasibility and acceptability of HBHCT among rural and urban populations. Studies on per client costs suggest the model is also economically viable [11,20]. Particularly promising is that HBHCT reaches populations with no prior testing experience [12-15,20] and identifies previously undiagnosed HIV-positive clients [12,14,16].

Based on current evidence, governments in sub-Saharan Africa are likely to expand and scale up HBHCT. Given the diverse nature of the sub-continent, and the fact that client characteristics are known to influence intervention uptake [13,14,19,21], the design of large scale national HBHCT interventions should draw upon experiences from a broad range of settings. While all findings may not be universally applicable, an understanding of important contextual issues beyond rates of uptake can help ensure success. Currently most of the literature on HBHCT comes from East Africa, where the socio-cultural context may be different from other settings. This paper reports on client characteristics and uptake of HIV testing within a home-based HIV testing intervention in a poor, rural South African community.

## Methods

### Study design and setting

This home-based HIV testing programme was implemented as part of a community-based cluster randomized-controlled trial comparing home-based HIV counselling and testing (HBHCT) to standard-of-care (mainly facility-based) HIV counselling and testing (HCT). The trial’s primary outcome was community level change in HIV testing rates; trial results will be published separately. With the aim of informing programme design for expansion and scale up, this paper, provides an in-depth description of the HBHCT intervention and its target population.

The HBHCT intervention described here was implemented in 8 trial and 11 non-trial clusters (Note: the primary trial included 16 randomised clusters - 8 control and 8 intervention – however, the intervention was expanded to 11 additional clusters in order to extend coverage for the national HIV counselling and testing campaign). The trial was registered (ISRCTN31271935) and granted ethical approval by the Ethics Committee of the South African Medical Research Council (Protocol ID: EC009-003).

The study was conducted in the Umzimkhulu sub-district, one of five local municipalities comprising the Sisonke District in Kwazulu-Natal (KZN). This area is one of the poorest in South Africa. It is characterized by dispersed rural settlements and out-migration to urban centres for work is common [22]. Thus a large proportion of households are female-headed [22,23]. Antenatal HIV prevalence in the Sisonke District is 35.2% [24] and a baseline survey conducted in the Umzimkhulu study clusters in 2008 found that only 32% of adult men and women had ever had an HIV test [25].

### Intervention training and implementation

The implementation team included 1 clinical nurse supervisor, 11 lay counsellors and 4 field supervisors. All staff members were females from the sub-district who spoke the local language, a mixed dialect of Zulu and Xhosa. All staff completed a 10-day nationally accredited course in HIV counselling and testing, and the counsellors spent four months gaining supervised testing experience at local health facilities. Additionally, they received training on couples counselling; family counselling; prevention of mother-to-child transmission; HIV and infant feeding; disclosure; TB/STI screening; and family planning. Supervisors participated in the general training and were further trained to monitor counselling and testing quality through formal counsellor observations and client exit interviews. Extensive community mobilization and interaction with local chiefs and leaders, as well as collaboration with the Sisonke District Department of Health through formal and informal meetings, were integral parts of study implementation throughout.

From September 2009 to January 2011, the lay counsellors systematically visited all unique households in the designated HBHCT intervention clusters. After seeking permission from the household head, they offered free HIV testing to all household members aged 18 years and older. Those aged 14–17 years were also offered testing provided they had parental or guardian consent. Counsellors were trained to encourage couples counselling and testing when appropriate. All clients gave oral consent for participation in the study, and written consent for the actual HIV testing, in accordance with local district procedures.

Lay counsellors read a translated information sheet to all potential clients, outlining study and testing procedures. They also gave basic HIV/AIDS education, after which clients were allowed to make a choice regarding participation. Clients who agreed were then met individually in a private room or section of the home, where survey administration, pre-test counselling, TB/STI screening, HIV testing, and post-test counselling took place.

The counsellors used the same rapid HIV test kits that were used by district health facilities during the study period – SD Bioline for screening and SENSA for confirmation of HIV-positive results. All clients were given a t-shirt stating “I know my status.” Distribution of t-shirts was aimed at boosting individual morale and raising community awareness about testing. During the testing session, counsellors used a standard checklist to verbally screen clients for signs and symptoms of tuberculosis (TB) or sexually transmitted infection (STI). Those with any signs or symptoms or who indicated a need for family planning were given a referral letter for further assessment at a health facility. Clients who tested HIV-positive were also given a referral letter to be taken to a local healthcare facility of their choice in order to obtain a CD4 count and other HIV-related services. Following testing, HIV-positive clients were contacted periodically by the counsellor to assess progress and access of needed health and social services. 

### Data collection and analysis

We partnered with an independent company ‘Mobenzi’ – making use of their mobile data collection platform Mobenzi Researcher. During the household visit and testing sessions, lay counsellors used cellular phones to obtain data on adult household membership (generally from the household head), client characteristics, and testing uptake (from individuals). No data was collected on the number or characteristics of children in each household as they were not in the target population for this study. Data captured in the field was then transmitted to a central web-based server, allowing it to be downloaded, monitored and analysed in real time. Data also passed through a validation filter, allowing errors (i.e. incorrectly entered study ID numbers or cluster names) to be identified and corrected in real time by study managers.

The software programme STATA version 11 was used to conduct analyses. Frequencies and proportions of HIV testing uptake, client characteristics, and reasons for refusal of testing were computed. Chi-square tests were performed to assess the association between client characteristics and testing uptake, as well as between gender and client characteristics among HBHCT testers. Chi-square tests were also performed to assess the association between client characteristics such as gender and age and reasons for refusal to test.

## Results

Lay counsellors visited 3,328 households. All but 3 household heads provided consent to enter the home and explain the study. The average household size was 2.1 adults. After repeated home visits to ensure full coverage, the counsellors met 6,757 people, which reflected 77.8% and 93.9% of all reported adult male and female household members. This also included 62 adult male and 110 adult female guests, and 123 males and 252 females aged 14–17 years.

Of the household members met, 28% were male and 72% female. The median age of males and females was 33 and 37 years, respectively. Table 1 shows characteristics of the study sample. Gender, age, and household size were all found to be associated with testing uptake, with a greater proportion of testers compared to non-testers being female and from smaller households. A greater proportion of non-testers were aged 25–49 years. Note that these three variables are the only data available for non-testers since people who refused testing generally also refused study participation, thus making it unethical to collect any more than the basic information from those who refused.

**Table 1:** Characteristics of people met by HBHCT lay counsellors

	**n**	**Testers %**	**Non-testers %**	***p*-value**
**Household Size**				
<3 adult members	3599	54.4	50.4	0.004
≥3 adult members	3136	45.6	49.6	
**Gender**				
Male	1896	26.3	33.5	<0.001
Female	4861	73.7	66.5	
**Age group**				
14–24	1985	31.5	24.5	<0.001
25–49	2538	34.9	48.9	
50–64	1229	19.3	15.8	
65+	898	14.3	10.8	
**Current marital status**				
Single	1916	37.7	---	---
Married	2123	41.7	---	
Co-habitating	176	3.5	---	
Widowed	818	16.1	---	
Divorced/Separated	53	1.0	---	
**Previous testing history**				
Tested previously	2164	42.6	---	---
Never tested	2912	57.4	---	
**HBHCT test result**				
HIV-positive	483	9.5	---	---
HIV-negative	4602	90.5	---	
Indeterminate	1	0.0	---	

**Figure 1:**
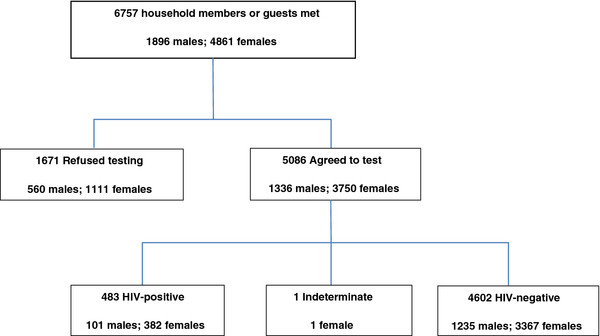
Flow chart of home-based HIV counselling and testing participants

Overall, 5,086 (75.0%) of the 6,757 people who were met, accepted a home-based HIV test. All but ten clients chose to receive their rapid test results immediately. While 3 received their results on another day, the remaining 7 did not receive them at all. Reflective of the underlying study population, nearly three-quarters of testers (73.7%) were female (Table 1). Only 3.7% of female testers were pregnant at the time of testing. Seventy-seven percent of females who were met, accepted testing compared to 70.0% of males. Four hundred and eighty-three (9.5%) clients tested HIV-positive, of which 46.4% reported never having tested previously. One client had an indeterminate test result. The remaining 4602 (90.5%) tested HIV-negative (Table 1). Figure 1 shows the breakdown of test acceptance and HIV-status, including gender.

Testers ranged in age from 14 to 98 years, with a median age of 37 years. All age groups were well represented; 31.5%, 34.9%, 19.3%, and 14.3% were aged 14–24, 25–49, 50–64, and 65+ years, respectively. With regard to marital status, 1,916 (37.7%), 2,123 (41.7%), and 818 (16.1%) of testers were single, married, and widowed, respectively. The rest were either co-habitating or divorced/separated (Table 1). While 1,625 (43.2%) of females reported being married, only 208 (12.8%) of them tested with a partner. In all, 229 couples received couples counselling and testing. The vast majority of couples (87.8%) were concordant negative; 4.8% were concordant positive. Seventeen (7.4%) couples were discordant; in 10 cases, the male was HIV-positive.

Fifty-seven percent (2,912) of home-based testers had never previously taken an HIV test (Table 1). Of those who had previously tested, the majority (63.2%) reported having only ever tested once. With regard to timing of previous testing, 58.3% reported having tested over a year ago, 16.4% reported having tested over 6 months but less than a year ago, 10.4% reported having tested between 4–6 months ago, and 14.9% had tested within the previous 3 months. The vast majority (93.0%) had previously tested at a clinic or hospital.

Table 2 shows the distribution of background characteristics among HBHCT testers. There were significant differences in the distribution of household size, age, marital status, previous testing history, and HBHCT test results between males and females who tested. In particular, compared to male testers, a larger proportion of female testers were from smaller households, older, married or widowed, HIV-positive, and had tested previously.

**Table 2:** Characteristics of testers by gender

	**n**	**Males %**	**Females %**	***p-value***
**Household Size**				
<3 adult members	2763	47.7	56.9	<0.001
≥3 adult members	2312	52.3	43.1	
**Age group**				
14–24	1602	37.1	29.5	<0.001
25–49	1773	31.5	36.1	
50–64	982	18.0	19.8	
65+	729	13.3	14.7	
**Current marital status**				
Single	1916	55.5	31.3	<0.001
Married	2123	37.6	43.2	
Co-habitating	176	3.1	3.6	
Widowed	818	2.9	20.8	
Divorced/Separated	53	0.8	1.1	
**Previous testing history**				
Tested previously	2164	23.6	49.4	<0.001
Never tested	2912	76.4	50.6	
**HBHCT test result**				
HIV-positive	483	7.6	10.2	0.016
HIV-negative	4602	92.4	89.8	
Indeterminate	1	0.0	0.0	

**Figure 2:**
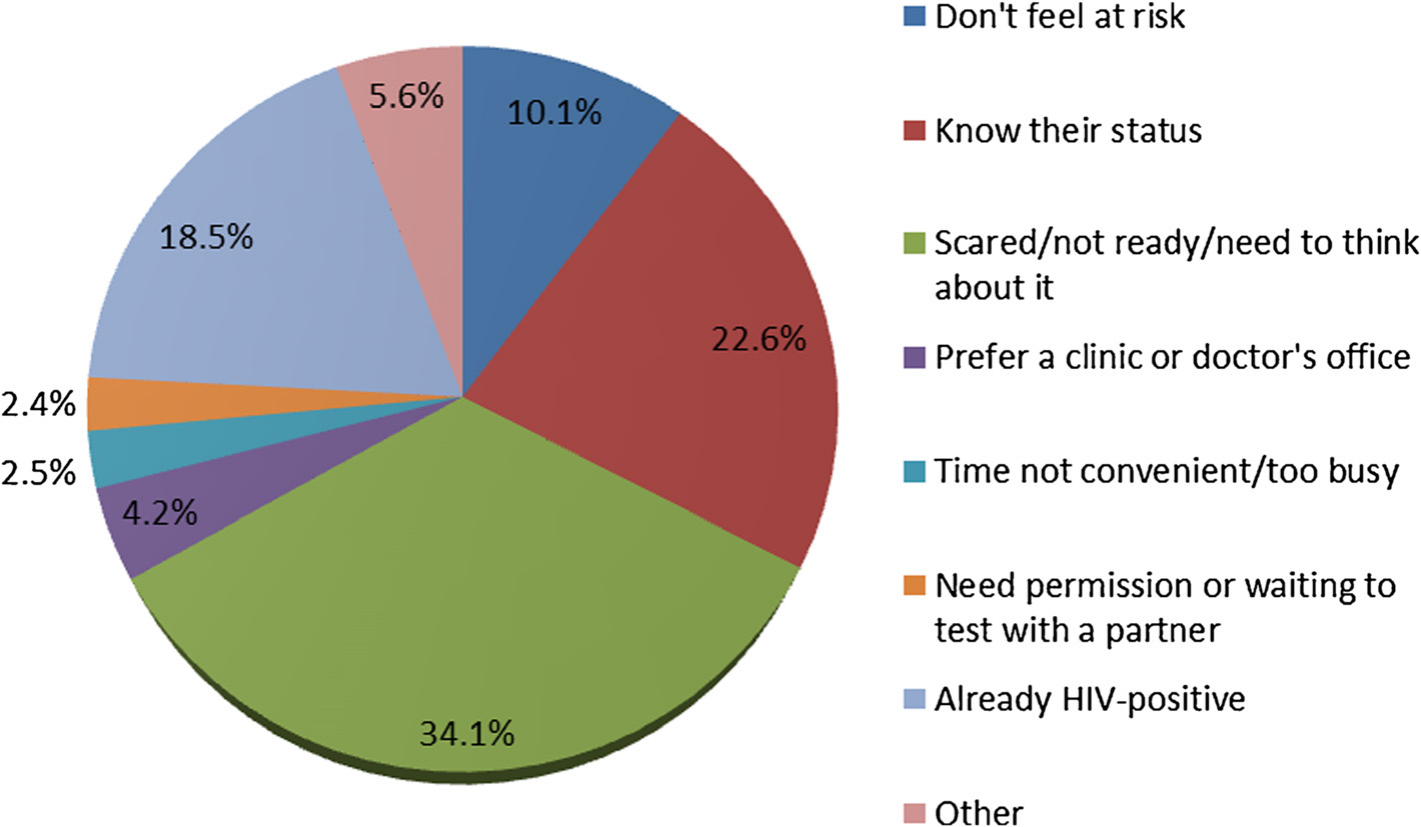
Reasons for refusal of home-based HIV counselling and testing

Of the 1,671 clients who refused HIV testing, 1,649 reported reasons for refusal (Figure 2). The most commonly reported reasons were: not being ready/feeling scared/needing to think about it (34.1%); knowing his/her status (negative or undisclosed) (22.6%), being HIV-positive (18.5%), and not feeling at risk of having or acquiring HIV (10.1%). There were significant differences in the distribution of reasons for refusal between males and females. Compared to females, a greater proportion of males reported: feeling scared/not ready/need to think about it (45.1% vs. 28.5%; *p* < 0.001) and not having time/being too busy (4.0% vs. 1.8%; *p* = 0.01). Compared to males, a greater proportion of females reported: already knowing their status (24.4% vs. 18.9%; *p* = 0.011); needing permission/waiting to test with a partner (3.1% vs. 0.9%; *p* = 0.005) and; being HIV-positive (22.6% vs. 10.4%; *p* < 0.001).

There was also a significant difference in the distribution of reasons for refusal to test between age groups. These are described for the four most commonly reported reasons. A greater proportion of those in the 14–24 age group (54.6%) reported not being ready to test compared to the older age groups: 25–49 (30.3%); 50–64 (27.6%); and 65+ (13.7%) (*p* < 0.001). A smaller proportion of the elderly aged 65+ (13.7%) reported already knowing their status compared to the younger age groups: 14–24 (20.4%); 25–49 (25.0%); and 50–64 (24.8%) (p = 0.008). A greater proportion of those aged 24–49 (28.0%) and 50–64 (14.6%) reported being HIV-positive, compared to those aged 14–24 (7.3%) and 65+ (2.4%) (*p* < 0.001). Fifteen percent of those aged 50–64 and 61.3% of those 65+ reported not feeling at risk of acquiring HIV compared to only 3.0% and 1.3% of those 14–24 and 25–49, respectively (*p* < 0.001).

## Discussion

This is the first study to report acceptability of home based HIV counselling and testing in rural South Africa. The intervention achieved a high uptake (75.0%) of home-based counselling and testing in a remote rural area. This is encouraging given that the baseline rate of testing in this area was only 32.0% [25] and that HIV-related stigma in this community is anecdotally high. We surmise that the intervention was successful because of the extensive mobilisation strategies used to introduce the project to local leaders and communities, high quality training which ensured that counsellors were confident offering their services, and community members’ trust in the counsellors’ skills and confidentiality.

The migratory patterns of this setting [22] may partially explain why we primarily met females in the home, and correspondingly, why they comprise the majority of those who tested. Furthermore, those with a marital partner largely tested alone, presumably because their partners were away. These circumstances may have critical implications. For example, females testing in the absence of male partners or authority figures may lead to potential negative social consequences [26] and could also impact disclosure, prevention, and care-seeking behaviour for those who test HIV-positive. While these issues were not formally studied within the context of this intervention, we did not receive any adverse reports following testing. However, further research is encouraged to inform future implementation and scale-up.

The gender stratified levels of uptake in our context are highly consistent with findings from other sub-Saharan African settings [15,17,21]. Although we reached a limited number of men overall, it is very encouraging that 70.0% of the males met did accept testing. Considering that only 17.0% of adult men had ever tested at baseline [25], it is unlikely that such a large proportion would have sought clinic-based services in the absence of HBHCT. Strategies to better reach men who are present in the community may include offering testing outside conventional working hours and during holiday periods, and employing male counsellors.

We found a relatively low level of sero-discordance among couples testing together. Though this is in contrast to findings from studies involving household members of TB or HIV-positive clients [16,17,27], it is within the range commonly found among general populations [28-30]. In our setting, couple testing was optional and it is possible there may have been self-selection of those with less risk. Furthermore, relatively few clients with a partner actually tested together, and only 7.5% of our HIV-positive clients tested with a partner. Thus our rates of discordance for this area may be underestimated.

The intervention reached a broad range of age groups. The majority of testers were in the reproductive age group of 14–49 years. This is promising since this population is generally the most sexually active and at risk of HIV acquisition. However, since high HIV prevalence and mortality are reported among older adults in sub-Saharan Africa [31,32], it is also important that our intervention reached this population. Additionally, in this traditional community the elderly serve as role models, and their participation may have encouraged others to do so as well.

It was also encouraging that the intervention reached a large proportion of people who had never previously tested or who had not tested recently, suggesting that increased access does lead to increased utilization, and that the HBHCT model may have mitigated other common barriers such as fear of stigma, lack of transport, or financial constraints. HBHCT also successfully identified a substantial proportion of previously undiagnosed HIV-positive clients, an important first step in ensuring timely access to care and treatment.

Our overall HIV prevalence of 9.5% is lower than expected when compared to the provincial HIV prevalence of 25.8% among those aged15-49 years in Kwazulu-Natal [5]. This may be explained in part by the fact that we tested a substantial number of clients who were well beyond the conventionally reported reproductive age range and that many clients who declined testing reported being HIV-positive. To test these theories, we recalculated HIV-prevalence for those aged 15–49 years, including refusals and classifying those who self-reported an HIV-positive status as HIV-positive, and classifying all others as HIV-negative. This resulted in an HIV prevalence of 14.3%, which is still well below the provincial rate. However, this may be explained in part by the fact that some of those who refused testing for other reasons or who were not met in the home, could have been HIV-positive. Further, it could be the case that this particular rural sub-community is less heavily affected by the epidemic than others in the province.

Males and females who were reached by the HBHCT intervention had distinctly different profiles. Compared to males who tested, a greater proportion of females who tested were from smaller households, older, married or widowed, HIV-positive, and had tested previously. These populations are likely to have distinctly different needs and concerns. For example older widowed women may need greater support related to acceptance of status while young single men may need more information about practising safer sex.

Very few people refused HBHCT because they preferred a clinical setting. This offers strong evidence of the HBHCT model’s acceptability in this community. Reasons for refusal varied significantly by age and gender. While these particular differences may be specific to our setting, they underscore the importance of better understanding target clients, and the potential need to tailor intervention messages and counselling for different sub-groups.

As previously stated, a sizeable proportion of non-testers reported their reasons for refusing as either being HIV-positive (18.5%) or already knowing their HIV status – negative or undisclosed (22.6%). The former fall out of the target group, and it is possible that even some clients in the latter group were HIV-positive but chose not to disclose their status. Combining these two groups, it means that 41.1% of non-testers were aware of their HIV status. This is comparable to the proportion of testers who reported ever having taken an HIV test prior to HBHCT (42.6%). However, amongst that group, over half (58.3%) reported having tested over a year ago. If the same applies for non-testers, knowing one’s status may not necessarily be a valid reason for refusal, particularly since the majority of those claiming to be aware of their status were in the 14–49 age group (80.3%) and likely to be sexually active. It is also possible that clients may have offered this reason to mask their true concerns or fears about testing. This implies that counsellors may need to give more attention to assessing clients’ reported reasons for refusal and further encouraging testing when appropriate.

From the sample of clients who refused testing, those who merit particular attention are the 34.1% who reported feeling scared or unready for a test. It is possible that their anxieties may stem from self-knowledge of risk behaviour, fear of the testing outcomes, stigma, or other reasons that were not easily voiced. To better motivate such clients it is important that we unpack and address the specific reasons behind their apprehension. The other group warranting closer attention is those who do not feel at risk of HIV infection. In our setting, this was most commonly reported by those in the 50–64 and 65+ age groups. As mentioned previously, given that HIV-prevalence and mortality are high among older age groups in sub-Saharan Africa [31,32], this may be a false perception that future interventions should address.

The findings of our study must be interpreted in light of important limitations. First, we do not have comparable background information on non-testers, which limited our ability to examine personal characteristics that are predictive of HBHCT uptake. Second, we did not ask people why they tested, and thus may have overlooked “hidden” influential factors. Third, we did not explore whether the HBHCT model might have led to unintended social consequences – for instance, clients feeling pressured to test or to disclose their status to those who also tested within the home, or women feeling un-empowered to share their results with partners not present at the time of testing. These potential outcomes are important and must be further studied. Fourth, since our study participants primarily included females, our findings may not be reflective of a more gender-balanced population. Fifth, we acknowledge that the provision of a t-shirt for people who agreed to test could have led some people to accept testing who otherwise might not have; however it is doubtful that a t-shirt alone would have been an influential factor for the majority of participants. Finally, the study was only conducted in one rural sub-district among those aged 14+ years, thus the findings may only be generalized to similar settings and adult populations.

This study also has several strengths. First, it involves a relatively large sample size. Second, it was conducted in an area with poor infrastructure and constraints typical of many rural areas in Africa, thus providing insight about the operational issues and challenges that may arise when scaling up in similar settings. Third, for the first time, we provide insight about the reasons for refusal of home-based testing, which may be as important as predictive socio-demographic background characteristics more commonly reported by other studies. Further, the gender and age-specific analyses offer an important understanding of client differences and their implications for programming.

## Conclusions

Adding to the current evidence base, these findings indicate that implementation of HBHCT in a rural area of South Africa is not only feasible, but also highly acceptable. Thus, it may be appropriate for scale-up in comparable settings. However, our findings also suggest that efforts to scale up should take account of important contextual factors and also consider the varying needs of sub-groups based on characteristics such as age and gender. Additionally, our study draws attention to important issues that may need to be addressed or further studied in order to ensure intervention success or mitigate potential harm. Overall, these findings can be used to inform programming and policy around the targeting, outreach, and design of interventions to scale up HBHCT in South Africa and other comparable settings.

## Competing interests

The authors declare that they have no financial or non-financial competing interests.

## Authors’ contributions

TD and DJ conceptualized the design of the cluster randomized controlled trial. RN, HT, TD, DJ and WZ designed the intervention. RN, HT, and TD developed data collection tools and designed the web-based data collection and management system. RN, HT, and WZ conducted staff training, managed study implementation and monitored data collection. RN conducted data analysis and drafted the original manuscript. TD, DJ, HT, and WZ critically revised the manuscript for important intellectual content. All authors read, contributed toward, and approved the final draft.

## Pre-publication history

The pre-publication history for this paper can be accessed here:

http://www.biomedcentral.com/1471-2458/12/824/prepub
